# Outer membrane vesicle contributes to the *Pseudomonas aeruginosa* resistance to antimicrobial peptides in the acidic airway of bronchiectasis patients

**DOI:** 10.1002/mco2.70084

**Published:** 2025-01-30

**Authors:** Yingzhou Xie, Yi‐Han Shi, Le‐Le Wang, Cheng‐Wei Li, Min Wu, Jin‐Fu Xu

**Affiliations:** ^1^ Shanghai Pulmonary Hospital, Institute of Respiratory Medicine, School of Medicine Tongji University Shanghai China; ^2^ Department of Pulmonary and Critical Care Medicine Huashan Hospital, Fudan University Shanghai China; ^3^ Department of Hepatobiliary and Pancreatic Surgery, The First Affiliated Hospital of Wenzhou Medical University, and Wenzhou Institute University of Chinese Academy of Sciences Wenzhou China; ^4^ Department of Respiratory and Critical Care Medicine Huadong Hospital, Fudan University Shanghai China

**Keywords:** 2‐heptyl‐4‐quinolone, antimicrobial peptides resistance, bronchiectasis, lactate, outer membrane vesicles, *Pseudomonas aeruginosa*

## Abstract

*Pseudomonas aeruginosa* is the predominant pathogen causing chronic infection in the airway of patients with bronchiectasis (BE), a chronic respiratory disease with high prevalence worldwide. Environmental factors are vital for bacterial successful colonization. Here, with sputa and bronchoalveolar lavage fluids, we determined that the concentration of airway antimicrobial peptide LL‐37 and lactate was elevated in BE patients, especially in those infected with *P. aeruginosa*. The in vitro antibacterial assay revealed the bactericidal activity of LL‐37 against the clinical *P. aeruginosa* isolates, which were dampened in the acidic condition. *P. aeruginosa* production of outer membrane vesicles (OMVs) enhanced in the lactate‐adjusted acidic condition. Transcriptomic analysis suggested that OMVs induce the hyperproduction of the chemical compound 2‐heptyl‐4‐quinolone (HHQ) in the bacterial population, which was verified by high‐performance liquid chromatography. The positively charged HHQ interfered with the binding of LL‐37 to bacterial cell membrane, potentiating the *P. aeruginosa* resistance to LL‐37. To our knowledge, this is a new resistance mechanism of *P. aeruginosa* against antimicrobial peptides and may provide theoretical support for the development of new antibacterial therapies.

## Graphical abstract

Airway lactate and LL‐37 enrich in the *P. aeruginosa*‐infected bronchiectasis patients. Outer membrane vesicles release of *P. aeruginosa* increases in the acidic environment. OMVs induce the overproduction of 2‐heptyl‐4‐quinolone (HHQ) in *P. aeruginosa*. HHQ protects *P. aeruginosa* from LL‐37 killing by inhibiting the binding to the membrane.

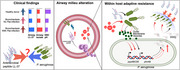

## 1 INTRODUCTION

*Pseudomonas aeruginosa* has been characterized as the major virulent pathogen persisting in the airways of patients with chronic respiratory diseases^1^, ^2^ especially bronchiectasis (BE) with increasing prevalence worldwide. The newly proposed model for BE development highlighted the key role of the chronic *P. aeruginosa* infection, which causes neutrophilic inflammation and the subsequent structural damage of the human airway^3^

Neutrophils primarily employ glycolysis to generate energy for the bactericidal activity, and the byproduct lactate can be released into the extracellular milieu, acidifying the local environment of the respiratory tract. We previously reported that airway acidification in BE patients promoted the type 1 interferon β response, impaired host defense against *P. aeruginosa* infection, and enhanced the intracellular survival of bacteria^4^ Therefore, the endo‐environment can be important in host‒pathogen interactions and its role needs to be further investigated.

Antimicrobial peptides (AMPs), including defensins, cathelicidins, and histatins, are another response of neutrophils to invading *P. aeruginosa*. LL‐37/hCAP‐18, the only cathelicidin produced by humans, is a crucial component of the innate immune system and functions in various respiratory diseases^5^, ^6^ Mature LL‐37 shows wide‐spectrum bactericidal activities against both gram‐negative and gram‐positive bacteria, such as *P. aeruginosa*, *Escherichia coli*, and *Staphylococcus aureus*.^7^ Our previous research showed that the transplantation of distal airway stem cells genetically modified to constitutively express human LL‐37 into mice can protect the lung from *P. aeruginosa* infection, indicating the potential of LL‐37 in innovating antibacterial therapies^8^ However, the bactericidal activity of LL‐37 can be affected by the intermolecular interaction within the host endo‐environment under pathological conditions^9^, ^10^ Additionally, bacteria have developed multiple strategies to resist AMPs, including proteome alterations in *Clostridium difficile* ^11^ proteolytic degradation in *Bacillus anthracis* ^12^ membrane structural remodeling in *Salmonella* ^13^ and via extracellular vesicle‐like structures produced by *Streptococcus pyogenes*.^14^

As mentioned above, *P. aeruginosa* is a predominant pathogen that colonizes with chronic respiratory diseases, where it is stressed by the host‐derived AMPs. We aimed to explore the factors contributing to *P. aeruginosa's* survival within the host and the underlying mechanism by which *P. aeruginosa* responds to the LL‐37 stress and gains resistance.

## 2 RESULTS

### 2.1 The antimicrobial peptide LL‐37 in airways was enriched in the BE patients with *P. aeruginosa* chronic infection

**FIGURE 1:**
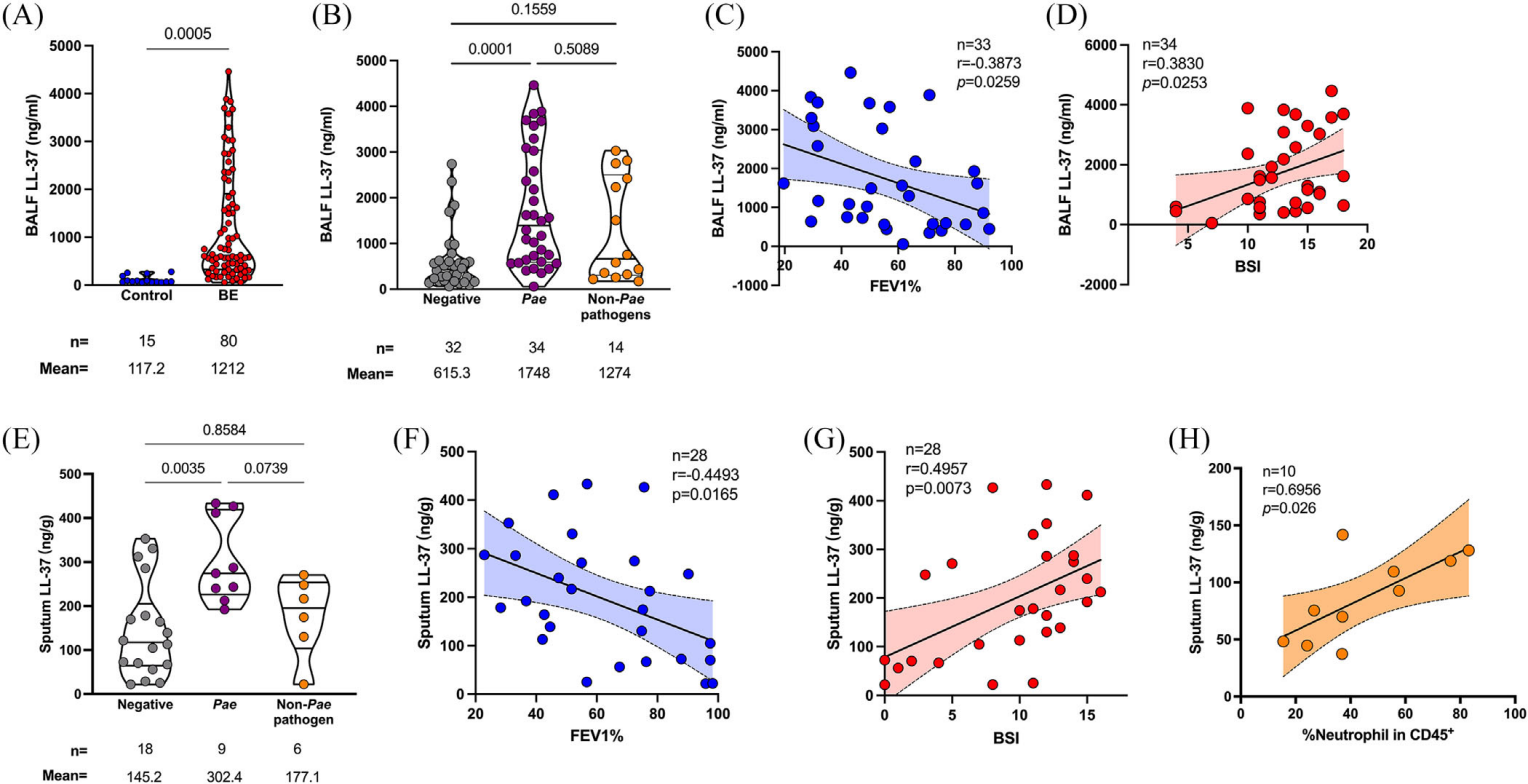
The antimicrobial peptide (AMP) LL‐37 increased in the biological fluids from bronchiectasis patients with *P. aeruginosa* chronic infection. (A) The LL‐37 concentrations in bronchoalveolar lavage fluid (BALF) of bronchiectasis (BE) patients and controls who were diagnosed without BE and underwent lung nodule surgery in our hospital in cohort 1. The BALF were from the contralateral lung. The comparison was performed with an unpaired Student t‐test. (B) Comparison of the LL‐37 concentrations in BALF between BE patients with different microbiology test results, which were identified by sputum culture. The comparison was performed with one‐way analysis of variance (ANOVA) with Welch's test. (C, D) show the correlation between BALF LL‐37 concentration to the lung function index percentage of forced expiratory volume in one second to the predicted value (FEV1%) and the BE severity index (BSI) in *P. aeruginosa*‐infected bronchiectasis patients, respectively. (E) Sputum LL‐37 concentrations of BE patients with different microbiology test results in cohort 2. The comparison was performed with one‐way ANOVA with Welch's test. (F, G) show the correlation between sputum LL‐37 concentration to the lung function index FEV1% and the BSI in 28 BE patients with BSI scores in cohort 2, respectively. (H) Correlation between sputum LL‐37 to the percentage of neutrophils in CD45+ immune cells isolated from the sputum. Ten sputa were randomly selected from cohort 2 to perform the analysis. *Pae* is short for *P. aeruginosa*. *p*‐Values less than 0.05 was considered statistically significant.

Oriol Sibila and co‐workers have reported that the LL‐37 concentration in the sputum of the European BE patients with *P. aeruginosa* infection was elevated, compared to those without infection or infected by other pathogens^15^ To validate and extend this observation, a total of 80 patients diagnosed with idiopathic BE and underwent bronchoalveolar lavage (BAL) at our hospital between May and December 2016 were enrolled in the present study (Tables S1 and S2). *P. aeruginosa* was identified as the predominant pathogen in the airways of this population by the sputum‐based bacterial culture, as it was isolated from 34 samples. Generally, the LL‐37 concentration was significantly higher in BAL fluid (BALF) from BE patients compared to the control (1212 ± 129.8 vs. 117.2 ± 20.74 ng/mL, *p* = 0.0005, data shown in Mean ± SEM, the same hereinafter) (Figure 1A). Taking the airway infection into account, the BALF LL‐37 concentration was determined even higher in the BE patients with *P. aeruginosa* chronic infection, compared to those with negative sputum culture (1748 ± 222.4 vs. 615.3 ± 115.8 ng/mL, *p* = 0.0001). Patients infected with germs other than *P. aeruginosa* also presented a higher BALF LL‐37 concentration than those without infection, but slightly lower than the patients with *P. aeruginosa* infection (Figure 1B). This difference cannot be observed in serum samples, revealing that the secretion of LL‐37 was a local immune response to bacterial infection (Figure S1). Further analysis with the *P. aeruginosa*‐infected patients showed a negative correlation between BALF LL‐37 levels and either the percentage of forced expiratory volume in one second to the predicted value (FEV1%) (*r *= 0.3873, *p* = 0.0259) or BE severity index (BSI) score (*r* = 0.3830, *p* = 0.0253) (Figure 1C,D). With sputum samples from a second cohort, which consisted of 33 patients with BE, we also found that the patients with *P. aeruginosa* were with higher LL‐37 concentration, compared to the patients with other pathogens isolation or negative sputum culture (Table S3 and Figure 1E). The correlation between sputum LL‐37 and either BSI or FEV1% was consistent with the BALF samples (Figure 1F,G). The quantity of neutrophils in the sputum positively correlated to the LL‐37 concentration, and this correlation can also be observed with the clinical blood test, suggesting the key role of neutrophils in the production of LL‐37 (Figure 1H and Figure S2).

Altogether, our clinical observations revealed that the colonization of *P. aeruginosa* correlates with the elevation of antimicrobial peptide LL‐37 in the airway of the patients with BE, and the neutrophil acted as a major producer of LL‐37. The contradictory coexistence of bacteria and antimicrobials suggested a resistance mechanism of *P. aeruginosa* to LL‐37.

### 2.2 The lactate‐adjusted acidic milieu dampened the bactericidal activity of LL‐37

**FIGURE 2:**
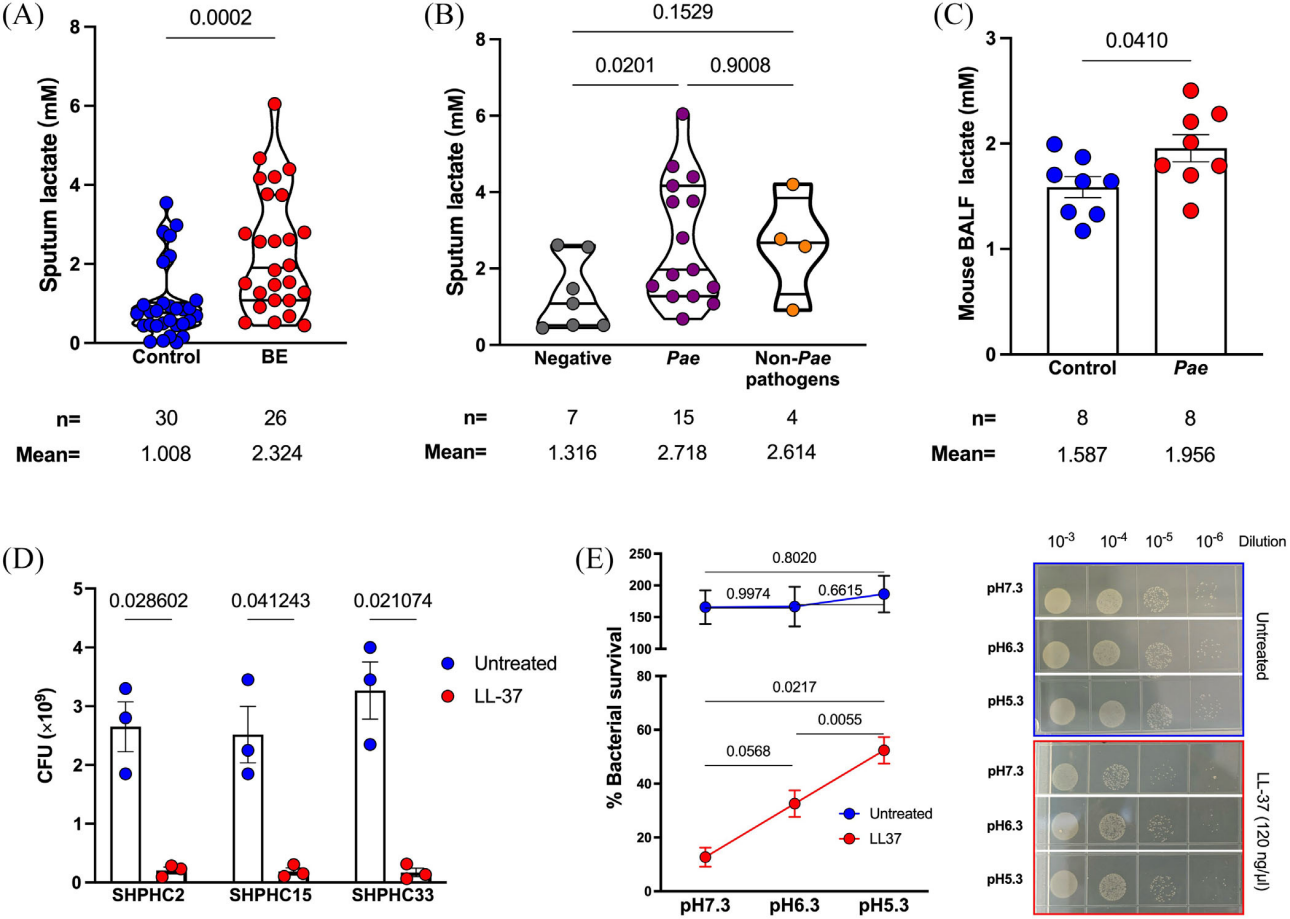
Lactate‐mediated acidification dampens the bactericidal activity of LL‐37 against *P. aeruginosa*. (A) Lactate concentrations of the sputa were collected from bronchiectasis (BE) patients and their relatives as controls in cohort 3. Induced sputa were collected when needed. The comparison was performed with an unpaired Student *t*‐test. (B) Comparison of the sputum LL‐37 concentrations between BE patients with different microbiology test results in cohort 3. A comparison was performed with one‐way analysis of variance (ANOVA) with Welch's test. (C) The lactate concentrations in bronchoalveolar lavage fluid (BALF) from mice with or without *P. aeruginosa* chronic lung infection. A comparison was performed with an unpaired Student *t*‐test. (D) The survivals of three clinical *P. aeruginosa* isolated from BE patients after the treatment with LL‐37 in vitro. Comparison between the untreated and LL‐37‐treated groups (120 µg/mL) was performed with an unpaired Student *t*‐test. (E) Percentages of survived clinical *P. aeruginosa* isolate SHPHC2 post‐LL‐37 treatment in the conditions with various pH adjusted by lactate. One‐way ANOVA with Welch's test was used for statistical analysis in both LL37‐treated and untreated groups, *p*‐values were indicated in the figure, and results with a *p*‐value less than 0.05 were considered statistically significant. The error bars plotted indicate the standard error of the mean.

Airway acidification is a pathological feature of chronic respiratory diseases.^16^, ^17^, ^18^ It also plays a role in the *P. aeruginosa*‐host interaction, as indicated by our previous research^4^ Thus, the acidic environment within the host can be a contributing factor to the *P. aeruginosa's* resistance to antimicrobial peptide. *P. aeruginosa* infection causes neutrophilic inflammation, and lactate is one of the main metabolites produced by neutrophils during bacterial infection. We sought to determine whether the lactate concentration in BE patients’ airways was elevated and whether it contributed to the within‐host resistance of *P. aeruginosa* to LL‐37. Sputum samples from a third cohort of patients with BE, who were diagnosed between October 2022 and June 2023 in our hospital, were collected to quantify lactate by colorimetry (Tables S4 and S5). The mean lactate concentration in the BE patient samples was 2.324 ± 0.2981 mM, which was significantly greater than that in the healthy control (1.008 ± 0.1722 mM; *p* = 0.0002). Consistent with the findings on LL‐37 concentration, the BE patients with *P. aeruginosa* chronic infection had elevated lactate concentrations (Figure 2A,B). We also observed that BALF from mice with chronic *P. aeruginosa* infection was of significantly higher lactate concentration, compared to the uninfected control (Figure 2C).

To determine the innate resistance of clinical *P. aeruginosa*, three isolates from BE patients with chronic infection were tested in vitro. Treatment with LL‐37 at a final concentration of 120 µg/mL efficiently killed all three bacteria within 1 h (Figure 2D). Therefore, there must be host factors contributing to the within‐host resistance to LL‐37 of *P. aeruginosa*, which was determined susceptible in vitro.

Next, we explored the effect of lactate‐mediated airway acidification on the resistance of *P. aeruginosa* to LL‐37. LL‐37 killing assays were performed in phosphate‐buffered saline (PBS) with 10% LB at various pH values, adjusted with lactate. The natural pH of PBS‐LB (v:v = 9:1) was determined to be 7.3. We adjusted the LL‐37 killing system at pH 6.3 and pH 5.3 to obtain a significantly acidic condition and confirm the impact of lactate‐mediated acidification on LL‐37 activity by the setup of a concentration gradient. After treatment with LL‐37 at 120 µg/mL, the clinical isolate *P. aeruginosa* SHPHC2 had a survival rate of 12.71% at pH 7.3, 32.6% at pH 6.3 (lactate final concentration: 0.006 M) and 52.4% at pH 5.3 (lactate final concentration: 0.012 M) (Figure 2E). The same phenomenon can also be observed in clinical isolates SHPHC15 and SHPHC33, revealing that lactate can significantly dampen the bactericidal activity of LL‐37 (Figure S3).

### 2.3 Outer membrane vesicles contribute to the *P. aeruginosa* resistance to LL‐37 in the acidic environment

**FIGURE 3:**
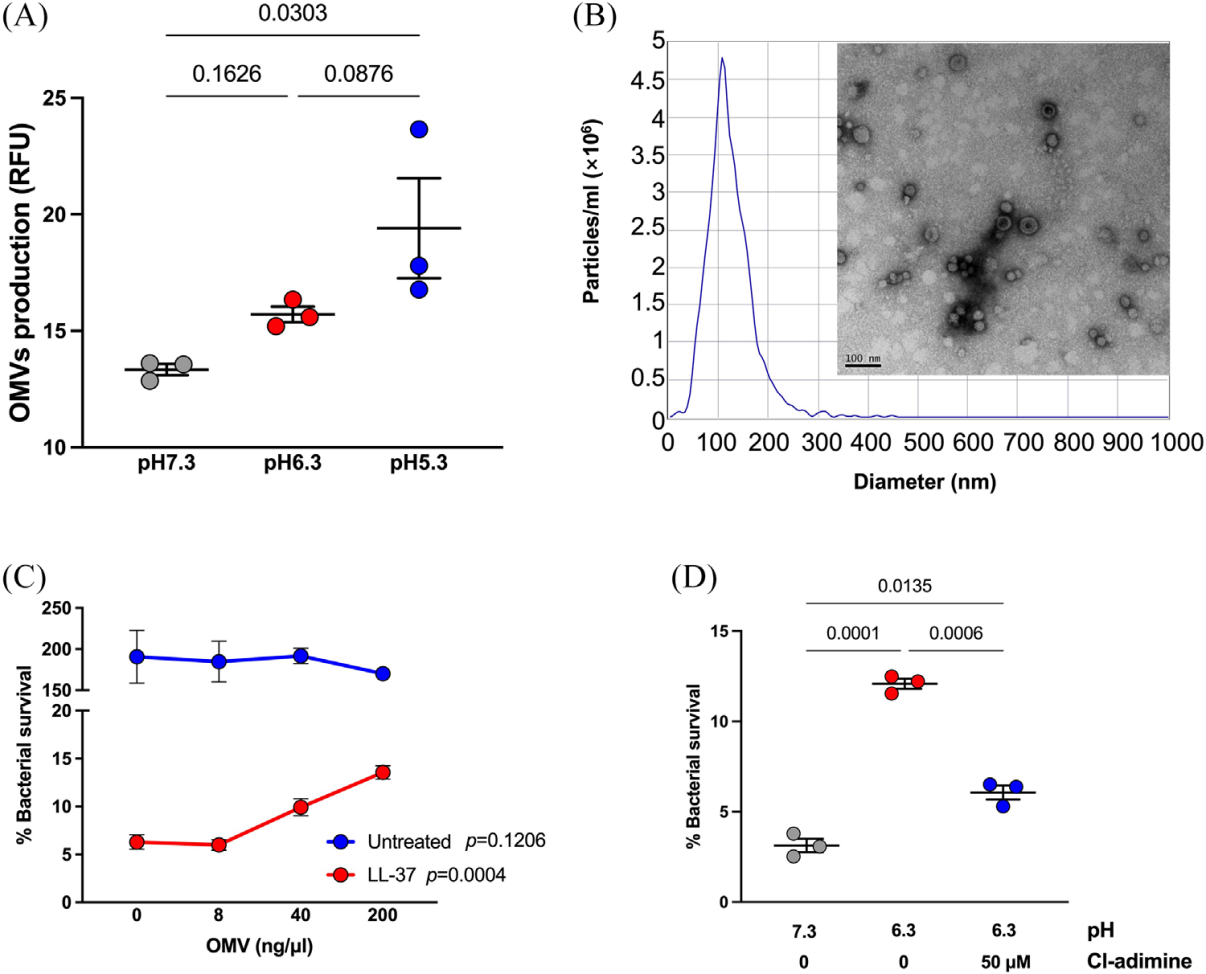
*P. aeruginosa* produces outer membrane vesicles (OMVs) to resist LL‐37‐mediated killing. (A) Quantification of OMVs produced by *P. aeruginosa* in the lactate‐mediated acidic environment by measuring FM 4‐64 fluorescence. One‐way analysis of variance (ANOVA) with Welch's test was used for statistical analysis. (B) Nanoparticle tracking analysis and transmission electron microscopy of OMVs isolated from *P. aeruginosa* overnight culture by ultracentrifugation. (C) Survival of *P. aeruginosa* PAO1 post‐LL‐37 treatment with the additions of OMVs of different dosages. The OMVs added were quantified by measuring the total proteins enwrapped. One‐way ANOVA with Welch's test was used for statistical analysis in both LL37‐treated and untreated groups and the *p*‐value was shown. (D) The role of OMVs in the acidic condition‐induced strengthening of *P. aeruginosa* LL‐37 resistance was determined by adding Cl‐adimine to the bacteria‐killing system. The final concentration of Cl‐diamine was indicated on the diagram. The differences between groups were statistically analyzed by using one‐way ANOVA with Welch's test, with the *p* values of multiple comparisons indicated in the figures. Results with a *p*‐value less than 0.05 were considered statistically significant. The error bars plotted indicate the standard error of the mean.

Outer membrane vesicles (OMVs) normally contain various kinds of biomolecules conferring functional versatility. By the quantification of total proteins, we previously reported that the OMVs generation was increased under the acidic conditions^4^. In this study, we re‐measured the OMVs production of *P. aeruginosa* under culture conditions with three different pH values, with the fluorescence dye FM 4‐64 binding to the lipid bilayer structure and emit red fluorescence. Experiments showed that OMV production increased along with the decline of pH (Figure 3A). We prepared OMVs from *P. aeruginosa* PAO1 cultured in LB media via ultracentrifugation. The purified OMVs exhibited a bilayer, hollow structure with a diameter ranging from 50–300 nm (Figure 3B). The isolated OMVs were added to the LL‐37 killing assay to mimic the elevation of OMV production under acidic conditions. Although the exogenous OMVs did not affect the bacterial growth, the addition of OMVs to the LL‐37 killing system significantly increased the survival of *P. aeruginosa* PAO1 (Figure 3C), in a dosage‐dependent manner. Additionally, inhibition of OMV release with Cl‐amidine, a protein arginine deiminase inhibitor, significantly reversed the reinforcing effect of lactate‐adjusted low‐pH conditions on the *P. aeruginosa* LL‐37 resistance (Figure 3D). Collectively, although the acidic environment can alter the LL‐37 conformation to dampen its bactericidal activity directly, our findings proved that excessive OMVs could also prevent *P. aeruginosa* from LL‐37 killing and was involved in the within‐host resistance of *P. aeruginosa* to LL‐37.

### 2.4 Overproduction of 2‐heptyl‐4‐quinolone mediated by OMVs inhibits the LL‐37 binding to *P. aeruginosa* cell membrane

**FIGURE 4:**
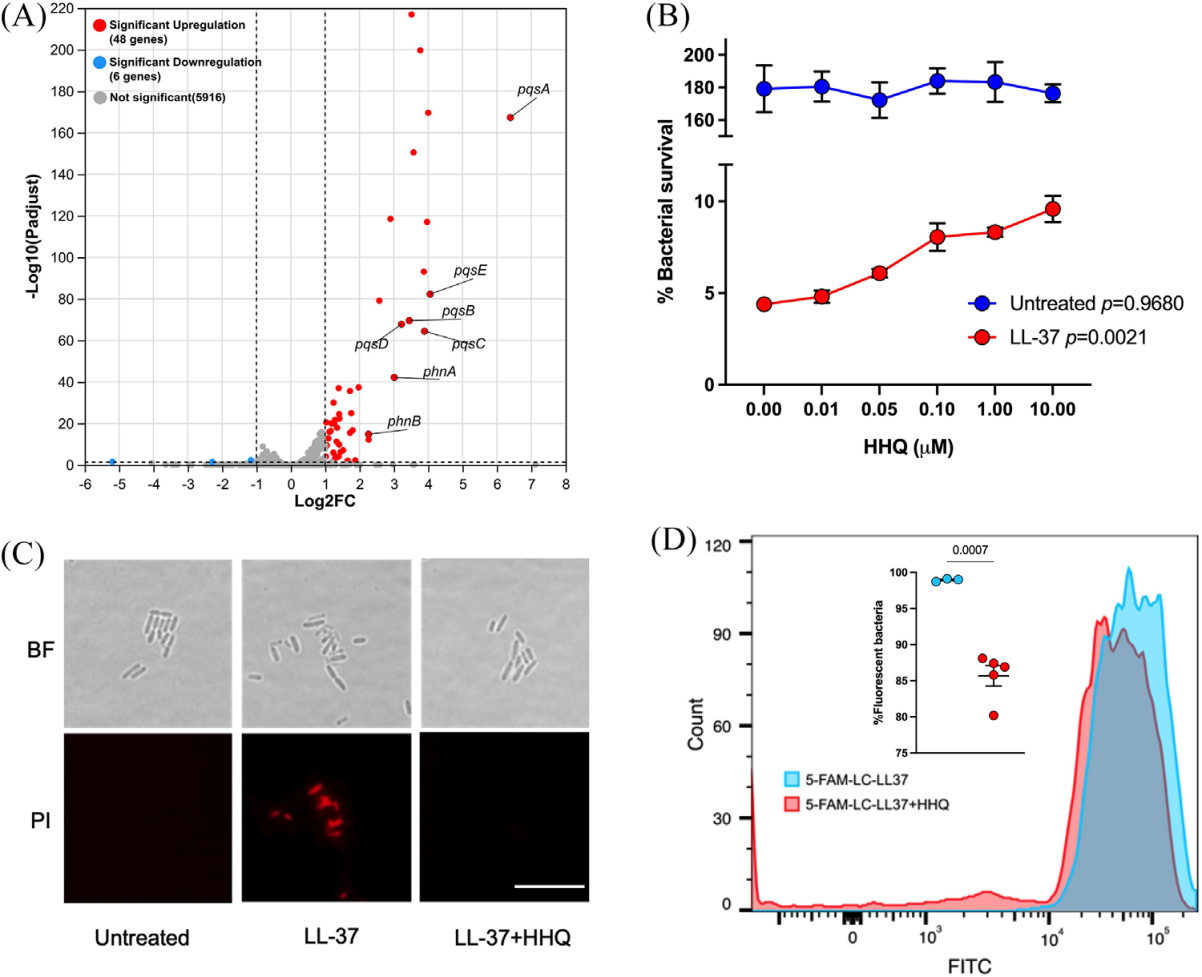
Outer membrane vesicles (OMVs) protected *P. aeruginosa* from LL‐37 killing by upregulating 2‐heptyl‐4‐quinolone (HHQ) production. (A) RNAseq analysis of the genes regulated by the addition of OMVs. Genes of interest are marked with the gene names from NCBI. (B) The effect of HHQ on the *P. aeruginosa* resistance to LL‐37 in vitro. A comparison was performed with one‐way analysis of variance (ANOVA) with Welch's test. (C) Representative microscopy images showing the survival of *P. aeruginosa* PAO1. Bacterial cultures were supplemented with 120 µg/mL LL‐37 or 120 µg/mL LL‐37 together with 0.1 µM HHQ and then placed in a static incubator at 37°C for 2 h before the images were acquired by an oil immersion objective. The scale bar indicates 10 µm. (D) Flow cytometry analysis of LL‐37‐absorbed *P. aeruginosa*. Commercially purchased 5‐FAM‐LC‐LL‐37 was used as the fluorescence‐tagged peptide and analyzed by using the FITC channel. The method of gating can be found in Figure S5. Student's *t*‐test was used for statistical analysis. *p* values were indicated on the figure and results with a *p* value less than 0.05 were considered statistically significant. The error bars plotted indicate the standard error of mean.

RNA sequencing was performed to determine the effect of OMVs on *P. aeruginosa* transcriptome regulation, which may account for the enhanced resistance to LL‐37. Forty‐eight genes were identified with upregulated transcriptions and six genes were downregulated significantly (Figure 4A). The gene cluster *pqsABCDE‐phnAB* for Pseudomonas quorum‐sensing signal molecule precursor 2‐heptyl‐4‐quinolone (HHQ) biosynthesis, which was constituted of two operons, was among the upregulated genes (Figure S4). Therefore, we presumed that HHQ could be the key molecule involved in the OMVs‐enhanced *P. aeruginosa* resistance to LL‐37. As expected, a supplement of HHQ significantly increased the survival of *P. aeruginosa* PAO1 after 1 h treatment with 120 µg/mL LL‐37, in a dosage‐dependent manner (Figure 4B).

By microscopy, fewer bacterial cells were stained with propidium iodide after 2 h of treatment with LL‐37 upon the addition of 0.1 µM HHQ, compared to those treated with LL‐37 only (Figure 4C). This indicated that HHQ can protect *P. aeruginosa* from LL‐37 killing at the single‐cell level. HHQ is a canonical signaling molecule involved in quorum sensing and biofilm formation. As the 1 or 2 h are not long enough for the biofilm formation, we speculated there were mechanisms other than biofilm by which the HHQ protected bacteria from LL‐37 bactericidal activity. LL‐37 is rapidly absorbed by bacteria before exhibiting antibacterial activity, via electrostatic absorption^19^ HHQ is a positively charged molecule due to the amine. Thus, it may interfere with the binding of LL‐37 to bacterial cell membranes. Flow cytometry showed that HHQ decreased the LL‐37‐bound *P. aeruginosa* from above 99% to 85%, by using fluorescence‐tagged LL‐37 (5‐FAM‐LC‐LL37) (Figure 4D and Figure S5). This is a new mechanism by which HHQ protects *P. aeruginosa* from antimicrobial peptide killing, which has never been reported before.

### 2.5 HHQ overproduction induced by OMVs is an innate response of *P. aeruginosa* to LL‐37 stress

**FIGURE 5:**
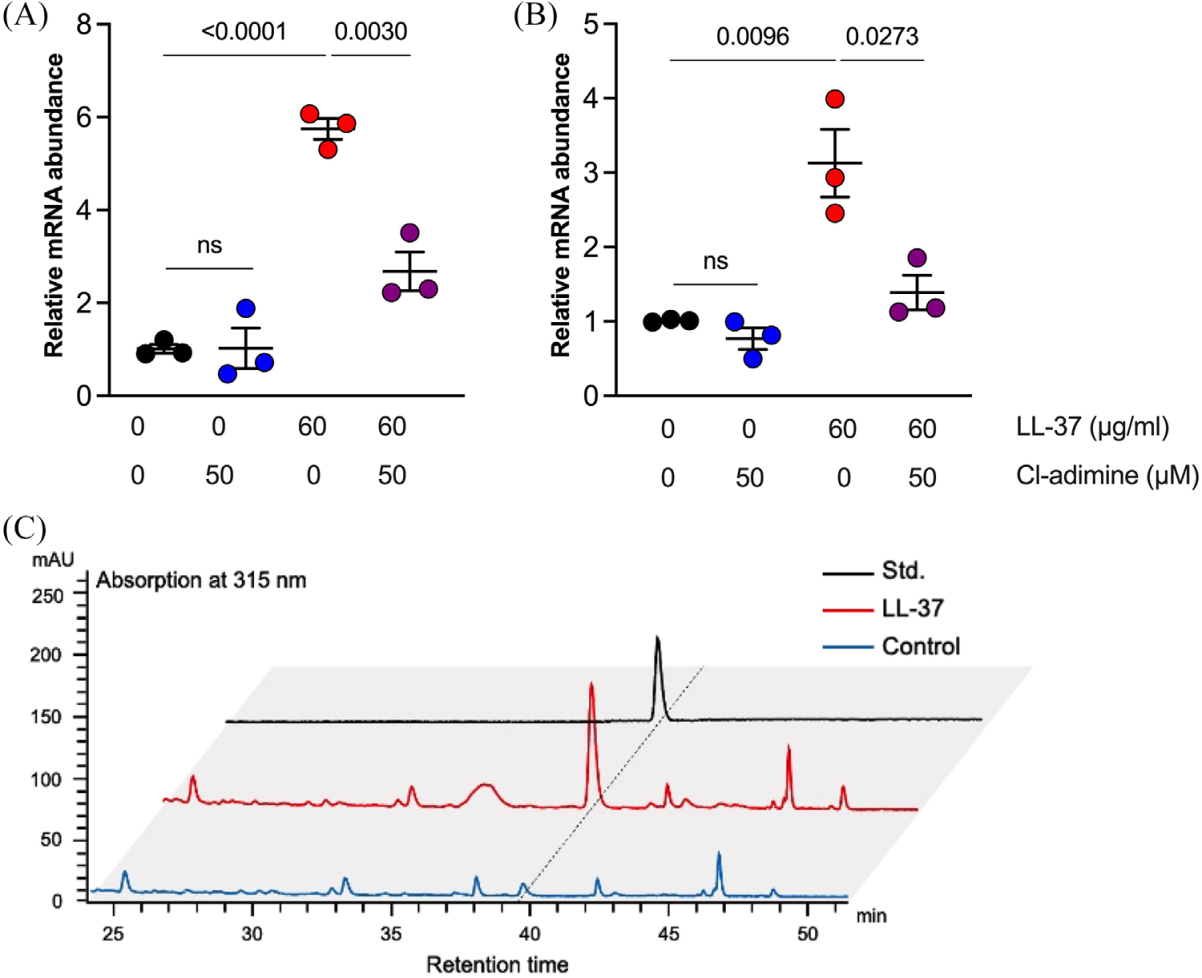
The overproduction of 2‐heptyl‐4‐quinolone (HHQ) was an innate response when *P. aeruginosa* was stressed by LL‐37. (A, B) The transcriptional levels of *pqsA* and *phnA* of *P. aeruginosa* PAO1 under different conditions. (C) The concentration of HHQ in the culture supernatant was determined via HPLC after the bacteria were stimulated with LL‐37. Commercially purchased HHQ was used as a standard for positioning the peak of interest and quantifying *P. aeruginosa*‐derived HHQ. Statistic of the HHQ production is shown in Figure S6. Student's *t*‐test was used for statistical analysis, *p*‐values were indicated on the figure, and results with a *p*‐value less than 0.05 were considered statistically significant. The error bars plotted indicate the standard error of mean.

As HHQ can increase the survival of *P. aeruginosa* challenged by LL‐37, we sought to determine its significance in the innate resistance to LL‐37 of this bacteria. The transcription of *pqsA* and *phnA* was obviously upregulated when *P. aeruginosa* PAO1 was stimulated with LL‐37 at 60 µg/mL, which showed no bactericidal activity in LB broth (Figure 5A,B). The HHQ concentration increase in the culture supernatant upon stimulation with LL‐37 was verified by high‐performance liquid chromatography quantification (Figure 5C and Figure S6). OMVs generation inhibition with Cl‐adimine significantly decreased *pqsA* and *phnA* transcription, indicating its vital role in the induction of HHQ production.

In conclusion, the upregulated HHQ production is an innate response and increases the resistance of *P. aeruginosa* to LL‐37 stress by reducing the binding of antimicrobial peptides to bacteria. OMVs act as key mediators in the promotion of HHQ production by LL‐37. The lactate‐mediated acid environment, which might be a result of bacterial infection, upregulates OMV generation and subsequently enhances *P. aeruginosa* resistance to LL‐37. This may explain how *P. aeruginosa* resists increased LL‐37 and establishes chronic infection in BE patients’ airways. Pre‐clinical clinical research with the currently available PqsD inhibitor or anti‐PqsR compound may benefit the BE patients suffering from recurrent *P. aeruginosa* infection^20^, ^21^ Development of drugs specifically targeting the bacterial OMVs production and quorum sensing molecules (including HHQ) can be a promising strategy to combat bacterial antimicrobial resistance.

## 3 DISCUSSION

Recruitment of neutrophils to the lung, acidification of airways, high secretion of antimicrobial peptides, and bacterial colonization are common pathophysiological alterations in not only BE patients but also patients with other chronic respiratory diseases, such as cystic fibrosis and chronic obstructive pulmonary disease (COPD)^22^, ^23^ However, studies on the crosstalk between these features are still lacking. By connecting these features together, we uncovered a novel mechanism that allows the susceptible *P. aeruginosa* to persist in the LL‐37‐rich milieu within the host. The airway acidification, which might be caused by *P. aeruginosa* infection, resulted in the increased generation of *P. aeruginosa* OMVs. OMVs further mediated the increase in bacterial HHQ production by upregulating the expression of the responsible genes. This molecule can reduce the binding of LL‐37 to the bacterial membrane, thus impairing LL‐37's antibacterial activity. The OMV‐mediated HHQ hyperproduction occurs naturally when *P. aeruginosa* is stressed by LL‐37; thus, this axis might constitute a novel mechanism of bacterial resistance deserving further investigation.

Mounting evidence reveals that OMV plays a versatile role in the bacterial life cycle. Our recent study showed that bacterial DNA coated within *P. aeruginosa* OMVs can trigger macrophages to secrete interferon β through the cGAS‐STING‐TBK1 pathway^4^ OMVs derived from *K. pneumoniae* have been shown to activate the host's inflammatory response via the promotion of macrophage proinflammatory pyroptosis^24^ OMVs also significantly contributed to bacterial antimicrobial resistance. β‐Lactamase and carbapenemase have been reported to be packaged into OMVs by various pathogens and released into the surrounding environment, resulting in enhanced bacterial resistance to antibiotics^25^, ^26^ Additionally, OMVs can be vesicles for the horizontal transfer of antibiotic resistance genes^27^ These existing reports, together with our findings that OMVs are key mediators of *P. aeruginosa* resistance to host‐derived antimicrobial peptides, indicate that OMVs can be ideal targets for new strategies to combat bacterial antimicrobial resistance.

HHQ and its derivative PQS are key factors in the quorum sensing of *P. aeruginosa*, and inhibitors of their synthesis are considered the next generation of antibacterial agents^20^, ^21^ However, the mechanism by which HHQ enhances antimicrobial resistance, other than by promoting biofilm formation, has rarely been identified. We showed that HHQ can reduce the absorption of LL‐37 by *P. aeruginosa* in this study, providing a new understanding of HHQ‐mediated antimicrobial resistance.

It has been reported that pH affects the secondary structure of LL‐37, which subsequently correlates with antibacterial activity^28^ This difference might also explain the low bactericidal activity of LL‐37 in the airways of BE patients, which has yet to be elucidated. Recently, LL‐37 was identified as an endogenous agonist of chloride intracellular channel 1 and contributes to Alzheimer's disease progression^29^ Thus, the role of increased LL‐37 in the airways of BE patients with pathological airway dilation, especially those with *P. aeruginosa* infection, needs to be further studied.

In this study, we uncovered novel crosstalk between the pathological features of BE patients and highlighted the important role of OMVs in *P. aeruginosa* resistance to the antimicrobial peptide LL‐37. However, most of the findings were derived from in vitro experiments. Although non‐invasive probes have been developed to detect the in situ lactate and pH in human tissues, neither of the new methodologies have been applied in clinical practice yet, and the absolute physiological concentration of airway lactate and LL‐37 can be hardly determined with biological fluids, including exhaled breath condensate (EBC), BALF and sputum^30^, ^31^ Therefore, the concentrations of LL‐37 and lactate may differ from their physiological concentration. Similarly, it is unlikely to quantify the *P. aeruginosa* OMVs and HHQ in biological fluids due to the super low abundance, therefore, the biological significances of these in the physiological conditions need to be further explored.

## 4 MATERIALS AND METHODS

### 4.1 Study design

This study was conducted by combining the clinical observations and in vitro experiments to explore the mechanism allowing *P. aeruginosa* can survive and establish chronic infection in the BE patients’ airway, where the antimicrobial peptide LL‐37 is enriched (Figure S7). Three clinical cohorts were involved to quantify the BALF LL‐37, sputum LL‐37, neutrophils in sputum, and sputum lactate. Cohort 1 consisted of BE patients admitted to Shanghai Pulmonary Hospital from May 2016 to December 2016 and the patients diagnosed without BE but underwent lung nodule surgery in our hospital in the same time period as controls. The BALF LL‐37 was measured in this cohort. As fresh‐collected sputa were required to measure the LL‐37, neutrophils, and lactate contained, two extra cohorts were enrolled. Cohort 2 consisted of BE patients admitted to Shanghai Pulmonary Hospital from June 2024 to July 2024. The data of sputum LL‐37 and neutrophil were generated with this cohort. Cohort 3 consisted of BE patients admitted to Shanghai Pulmonary Hospital from February 2023 to June 2023 and their relatives as controls. The sputum lactate was quantified with this cohort. The protocol of this study was approved by the ethics committees of Shanghai Pulmonary Hospital with the approval numbers K17‐127 and K22‐336, and all patients have provided signed informed consent. All the cohorts were further divided into subgroups based on the sputum microbiology test. Besides *P. aeruginosa*, the microorganisms isolated from the BE patients’ sputa were *Candida albicans*, *Viridans streptococci*, *Neisseria spp*., *Klebsiella pneumoniae*, *E. coli*, non‐tuberculous Mycobacteria, *Aspergillus spp*., *Stenotrophomonas maltophilia*, *Aeromonas hydrophila*, *Candida glabrata*, and *Raoultella planticola*.

The in vitro LL‐37 killing assays on *P. aeruginosa* clinical and laboratory isolates with various chemicals and purified OMVs were performed to reveal the vital role of OMVs in *P. aeruginosa's* adaptive resistance to antimicrobial peptide LL‐37. RNA sequencing was utilized to the regulatory mechanism of OMVs on *P. aeruginosa* to enhance the resistance to LL‐37.

### 4.2 The criteria for BE patient's enrollment

All enrolled patients were required to meet the inclusion criteria as follows: (i) Patients aged ≥18 years; (ii) Patients provided signed informed consent; (iii) The patient was clinically diagnosed with idiopathic BE.

The diagnosis of BE was based on the 2019 British Thoracic Society guidelines^32^ Symptoms included chronic cough with expectoration, with or without intermittent hemoptysis, and chest CT indicating BE. Patients with known causes of BE, such as primary ciliary dyskinesia (PCD), diffuse panbronchiolitis (DPB), and allergic bronchopulmonary aspergillosis (ABPA), were excluded. The additional exclusion criteria for both groups were as follows: (i) Individuals with a history of respiratory diseases such as cystic fibrosis, ABPA, asthma, alpha‐1 antitrypsin deficiency, pulmonary tuberculosis, COPD, lung cancer, or interstitial lung disease, as well as conditions such as gastroesophageal reflux disease and atopic diseases; (ii) Participants currently using inhaled medications, including corticosteroids, antibiotics, or bronchodilators; (iii) Patients who participated in any interventional clinical trials in the past 3 months; (iv) A history of smoking or alcohol abuse disqualifies potential participants.

### 4.3 Bacteria, primers, and culture conditions

The information on the bacteria and primers for the quantitative reverse transcript polymerase chain reaction used in this study were listed in Tables S5 and S6. All the bacteria were cultured on the LB agar plates or in the LB broth, with supplements of OMVs or chemicals, depending on the experimental purpose. All the bacteria were incubated at 37°C and the shaking speed was set to 220 rpm if needed.

### 4.4 Clinical sample collection and quantification

The detailed inclusion and exclusion criteria are provided in the supplementary materials. The study cohort was further divided according to the presence or absence of *P. aeruginosa* infection based on microbiological analysis of sputum.

The collection and processing of BALF were conducted using a standardized approach with fiberoptic bronchoscopy with a total of 20 mL sterile 0.9% saline. The acquired were promptly filtered through sterile gauze to meticulously remove any excess debris or mucus. This was followed by centrifugation of the samples at 3000 rpm for 5 min. The supernatant was carefully separated and preserved at a temperature of ‐80°C before assay. For the measurement of LL‐37 concentration in these BALF samples, the enzyme‐linked immunosorbent assay (ELISA) kit (Hycult Biotechnology, catalog # HK321) was used. BALF from the normal side of the lung of non‐BE patients diagnosed with lung nodules was designed as a healthy control.

To collect the sputum sample, patients were instructed to rinse their mouths with saline for approximately 3 min to reduce contamination from food residues and other substances. For sputum collection, especially when patients or healthy controls produce minimal sputum, sputum induction is carried out using a nebulizer. This procedure involved adding approximately 6–8 mL of 0.9% saline solution to a disposable nebulization bottle, which was then connected to a nebulization mask. The patient, fitted with the mask, is subjected to nebulization for 10–20 min until sufficient sputum is produced.

Freshly collected sputum should be processed within 2 h. For one gram of sputum, two ml of PBS containing 0.1% (w/v) dithiothreitol were added and mixed. The mixture was incubated in a 37°C water bath for 15 min and then centrifuged at 500×g for 5 min. The supernatant was used for the quantification of LL‐37 and lactate, by ELISA kit (Hycult Biotechnology) and Lactate Assay Kit (Nanjing Jiancheng Bioengineering Institute, catalog A019‐2‐1), respectively. The pellet was resuspended in PBS, processed with a 70 µm‐sized filter, and washed with PBS twice. Cells were stained with Fixable Viability Stain 510 (BD Biosciences, cat. 564406), washed, stained with BD Pharmingen PerCP‐Cy 5.5 Mouse Anti‐Human CD45 (cat. 564105) and BD Horizon BV421 Mouse Anti‐Human CD16 (cat. 562874), and analyzed using BD FACSAria III.

### 4.5 LL‐37 killing assay on *P. aeruginosa*

*P. aeruginosa* clinical isolates SHPHC2, SHPHC15, and SHPHC33 and the lab isolate PAO1 were used to perform the LL‐37 killing assay depending on different purposes. Overnight cultures of bacteria were diluted at 1:100 into fresh LB broth and further cultured for 4 h at 37°C in a shaking incubator at 220 rpm. Then 1 mL of subculture was washed with sterile PBS twice, and the pellet was resuspended in 100 µL PBS‐LB mixture (PBS:LB = 9:1, v/v). Less than 5 µL stock solution of chemicals or isolated OMVs were added to the system, depending on the different experimental objectives. The final concentrations of LL‐37 (Tocris Bioscience, cat. 5213), *P. aeruginosa*‐derived OMVs, HHQ (TargetMol, cat. T19713), PQS (TargetMol, cat. T38373) and Cl‐adimine (MedChemExpress, cat. HY‐100574) were stated in the main text. The mixture was placed statically at 37°C for 1 h. The bacteria were washed 3 times with pre‐warmed PBS before plating to remove the chemicals that may affect the bacterial growth. Serial dilution was performed and 10 µL of each dilution was spotted on the LB agar for the CFU counting the next day.

### 4.6 Isolation and quantification of outer membrane vesicles

*P. aeruginosa* PAO1 was streaked on the LB agar medium and incubated at 37°C overnight. One single colony was picked and inoculated into 4 mL LB broth for overnight shaking culture. Then the whole culture was poured into 500 mL fresh LB broth for amplification. To investigate the impact of pH on OMV production, the LB broth was supplemented with lactate to reach a pH of 6.3 or 5.3. The culture was then applied to a centrifuge at 12,000×g for 15 min at 4°C. Afterward, the supernatant was collected, filtered through a 0.45 µm pore, and then ultracentrifuged for 3 h at 65,000×g and 4°C (SORVALL WX+ ULTRA SERIES centrifuge; Thermo Scientific). The supernatant was discarded, and the remaining pellet was washed with cold sterile PBS, followed by another round of ultracentrifugation under the same conditions. Finally, the pellet was resuspended in 500 µL of sterile PBS and stored at ‐80°C for future use. The purified OMVs were analyzed with transmission electron microscopy (TEM) and nanoparticle tracking analysis.

For the quantification of OMVs with FM 4‐64 (ThermoFisher, cat. 13320), the bacterial cultures in different conditions were centrifuged at 12000×g for 15 min. The supernatants were filtered with the 0.22 µm pore size filter. FM 4‐64 at a final concentration of 5 µM was added to the supernatant to be assayed. The mixture was mixed gently and incubated in the dark for 15 min. The fluorescence intensity was subsequently measured using a fluorescence spectrophotometer according to the instructions of FM 4‐64.

### 4.7 Construction of a mouse model with chronic *P. aeruginosa* airway infection

The mouse model was constructed as per the protocol described by Hu et al., with minor modifications^33^ Briefly, the LB broth was used instead of TSA, and we used agarose rather than agar in this study. For particle size analysis, 20 µL of PAO1 agarose beads were transferred into a 10 cm dish with 10 mL of PBS and applied to microscopy with the Zeiss Axio Observer inverted microscope. The preparation was considered successful if the size of over 90% of particles was between 100 and 200 µm, measured by the microscopy. Bacterial concentration was determined by counting the colonies on CN agar (a selective medium for *P. aeruginosa*) after destroying the agarose beads by homogenization and serial dilution. 6–8‐week‐old female C57BL/6 mice were anesthetized with pentobarbital sodium and were intratracheally infected with PAO1 agarose beads (2 × 10^6^ CFU in 50 µL PBS). The model was considered successful if over 1000 CFU of *P. aeruginosa* could be isolated from the whole lung on the 10th day post‐infection.

The BAL fluids were collected from mice as per the protocol published by Adhikari et al.^34^ Briefly, the mice with *P. aeruginosa* chronic infection or in the control group were anesthetized and placed in the supine position. The skin on the neck was opened and a small incision was made on the trachea. A 1 mL‐sized syringe with a blunt needle was inserted into the trachea and the lung was lavaged with 0.5 mL sterile PBS twice. The fluids were then collected for further measurements.

### 4.8 Data analysis

The quantitation of LL‐37 in BALF, lactate in sputum, and the HHQ in the supernatant were calculated with the fitted standard curve. All the comparisons between the two groups were conducted by using the unpaired student *t*‐test. Comparisons between more than two groups were analyzed using one‐way analysis of variance with Welch's test. *p* value less than 0.05 was considered statistically significant.

## AUTHOR CONTRIBUTIONS

Y.X., Y.H.S., and L.L.W. contributed equally to the work. J.F.X. and Y.X. conceived the project and designed the experiments. Y.X., Y.H.S., and L.L.W. performed the OMVs isolation and the LL‐37 killing assay on *P. aeruginosa* under various conditions. Y.H.S. and C.W.L. collected the clinical samples and did the quantification of LL‐37 and lactate. Y.H.S. and L.L.W. did the molecular experiments, infection of a mouse model, and statistical analysis. Y.X., M.W., and J.F.X. drafted, reviewed, and revised the manuscript. All the authors have full access to the data of this study and approved the final version of the submission.

## CONFLICT OF INTEREST STATEMENT

The authors declare no conflict of interest.

## ETHICS STATEMENT

The collection of clinical samples was approved and supervised by the ethics committee of Shanghai Pulmonary Hospital, with the numbers K17‐127 and K22‐336. Written informed consent was obtained from all participants. Experiments on mice were approved by the ethics committee of Shanghai Pulmonary Hospital with the number K22‐336.

## Supporting information



Supporting Information

## Data Availability

Data that support the findings of the present are provided within the manuscript and the appendix. The concrete data can be acquired from the corresponding author with the request via email. Raw data from the RNA sequencing for OMVs‐treated *P. aeruginosa* PAO1 has been deposited in the Sequence Read Archive of the National Center for Biotechnology Information under the BioProject PRJNA1070371, and accession numbers SAMN39643812 and 39643813.
