# Supplementary material for: Outer membrane vesicle contributes to the Pseudomonas aeruginosa resistance to antimicrobial peptides in the acidic airway of bronchiectasis patients
Source: MedComm (2020). 2025 Jan 30;6(2):e70084. doi: 10.1002/mco2.70084 (PMC11782972; doi:10.1002/mco2.70084)
Supplement: Supplementary file 1 — Supporting Information [file MCO2-6-e70084-s001.docx]

**Supporting information for**

**Outer membrane vesicle contributes to the *Pseudomonas aeruginosa* resistance to antimicrobial peptides in the acidic airway of bronchiectasis patients**

Running title: OMVs contribute to *P. aeruginosa* resistance to AMP

Yingzhou Xie^a,b^, Yi-Han Shi^a,b^, Le-Le Wang^a,b^, Cheng-Wei Li^c^, Min Wu^d^, and Jin-Fu Xu^a,b^

^a^Department of Respiratory and Critical Care Medicine, Shanghai Pulmonary Hospital, School of Medicine, Tongji University, 507 Zhengmin Road, Shanghai, China

^b^Institute of Respiratory Medicine, School of Medicine, Tongji University, 507 Zhengmin Road, Shanghai, China

^c^Department of Pulmonary and Critical Care Medicine, Huashan Hospital, Fudan University, Shanghai, China

^d^Department of Hepatobiliary and Pancreatic Surgery, The First Affiliated Hospital of Wenzhou Medical University, and Wenzhou Institute, University of Chinese Academy of Sciences, Wenzhou, China

Y. Xie, Y-H. Shi and L-L. Wang contributed equally to this work.

**Corresponding author**:

Prof Jin-Fu Xu, MD

507 Zhengmin Road, Shanghai, China 200433

Email: jfxu@tongji.edu.cn

Tel: +86(0)13321922898

# Supplementary Methods

## RNA sequencing and data analysis

*P. aeruginosa* PAO1 was cultured in shaking incubator set at 37℃ and 220 rpm for overnight. Then the culture was diluted in fresh LB broth at a ratio of 1:100. OMVs purified by ultracentrifuge were added to make the mixture contain OMV-containing proteins at a final concentration of 200 μg/ml. Bacteria with/without OMVs treatment were cultured in a shaking incubator set at 37℃ and 220 rpm for 6 hours and harvested by centrifuge at 9000×g for 5 min. Pelleted bacteria were resuspended in Trizol (ThermoFisher, cat. 15596018CN). Bacterial RNA was extracted, processed and sequenced by Shanghai Majorbio Biotech with Illumina NovaSeq X plus. The generated reads were mapped to the whole genome sequence of *P. aeruginosa* PAO1 to identify the transcriptional levels of annotated genes. Genes with a two-fold quantity changes between OMVs-treated bacteria and control and an adjusted p value less than 0.05 were considered with significantly differential expression. Differentially expressed genes within the identical transcriptional unit (operon) were predicted using Rockhopper^1^. Pseudomonas genome database was referred to identify the genes or gene clusters executing the same or related biological functions^2^.

## Quantification of HHQ Production

The overnight culture of *P. aeruginosa* PAO1 from a single colony was inoculated into fresh LB broth at a ratio of 1:100. Bacteria was then incubated at 37°C in a shaking incubator for overnight. The culture was centrifuged at 9000×g for five minutes, the resulted supernatant was discarded, and the bacteria were resuspended in fresh LB medium to an OD600 of 0·8 and divided into two parts. One part was used for the stimulation experiment, in which LL-37 with the final concentration of 60 µg/ml (determined as the sublethal dosage in LB broth) was added. The other part was used as control group, in which water of equal volume was added. All those mixtures were incubated for another two hours at 37°C. Subsequently, the cultures were harvested by centrifugation at 10000×g for ten minutes. The resultant supernatant was collected and extracted with equal volumes of EtOAc for three times. The resultant supernatant was combined and dried under reduced pressure. After that, one milliliter of methanol was used to dissolve the mixtures and the mixture was then filtered through a 0·22 µm nylon membrane before concentrated into 50 µl. Fifteen microliters of each sample were injected for quantitative analysis by HPLC.

The commercially available HHQ standard of 1 mg was dissolved with methanol of 385·6 µl, affording the stock solution of 10 mM. HPLC analysis was carried out on an Agilent 1260 HPLC system using an Agilent ZORBAX SB-C18 column (5 μm, 4·6 × 250 mm). For the detection optimization, HHQ of 100 µM was used. The column was equilibrated with 85% (v/v) solvent A (H_2_O with 0·1% formic acid) and 15% (v/v) solvent B (acetonitrile) and developed with a linear gradient (5-30 min, from 15% B to 50% B, 30-35 min, from 50% B to 60% B, 35-40 min, from 60% B to 80% B, 40-45 min, from 80% B to 100% B) and then kept 100% B for 5 min at a flow rate of 0·6 ml/min and UV detection at 210 nm, 245 nm, 254 nm, 274 nm, 388 nm and 420 nm. The standard HHQ exhibited characteristic absorption peak at 234 nm and 315 nm with the retention time of 40 min. The subsequent analysis of HHQ was conducted with UV detection at 210 nm, 234 nm, and 315 nm. The standard curve of HHQ with different final concentration of 5 µM, 10 µM, 20 µM, 50 µM, 100 µM, 250 µM, and 500 µM. Fifteen microliters of HHQ with different concentration was injected and analyzed by HPLC for twice. The standard HHQ formula was obtained about the absorption area (at 234 nm) corresponding to the final concentration. The concentration of HHQ in samples of stimulation group and control group was calculated according to above formula.

# Supplementary Figures

**
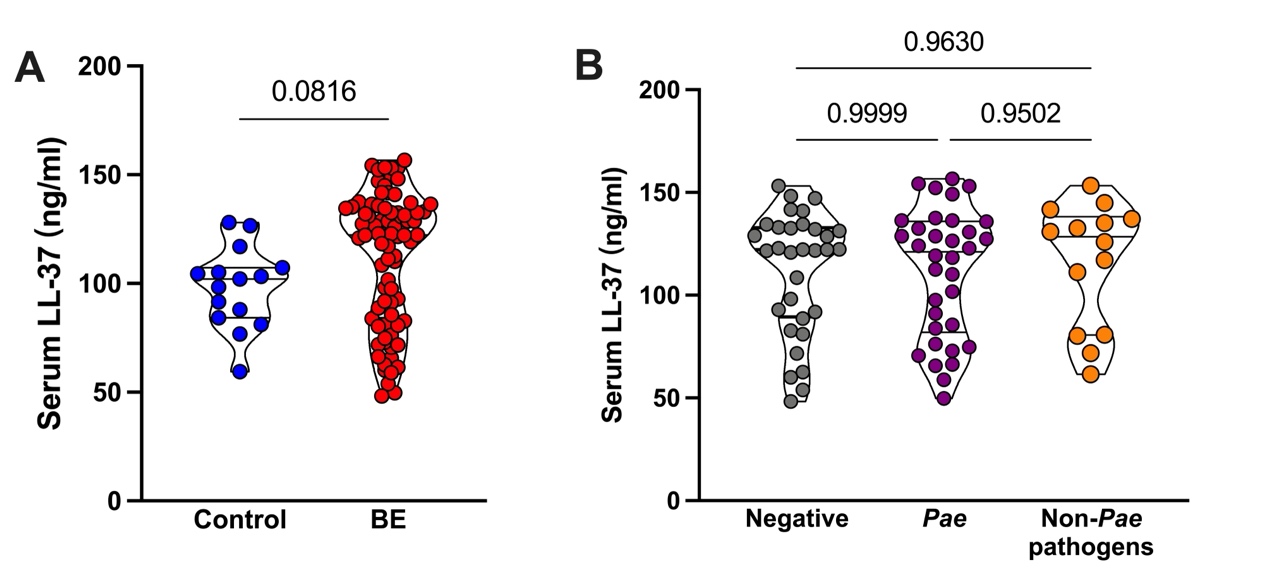
**

## Figure S1. The comparison of LL-37 concentrations of serum sampled from cohort 1. LL-37 was measured by using ELISA kit from Hycult Biotechnology. (A) Serum LL-37 concentration was compared between samples from heathy control and those from BE patients. The difference was analyzed with unpaired Student t test. (B) The comparison of serum LL-37 between BE patients with different microbiology test results. One-way ANOVA with Welch’s test was used for statistical analysis and multiple comparisons were performed to generate the *p* values. *p* value less than 0.05 was considered statistically significant. BE and *Pae* are shorts for bronchiectasis and *P. aeruginosa*, respectively.

**Figure S2. Sputum LL-37 positively correlates to the blood neutrophils in patients with BE.** The abundance of blood neutrophils was determined by the regular blood test. Sputum LL-37 was quantified in the freshly collected sputa treated with DTT, by ELISA. The correlation analysis was performed using GraphPad Prism 10.

**Figure S3. Acidic environment increased the survival of clinical *P. aeruginosa* isolates after LL-37 killing *in vitro.*** The dots in red, purple and blue show the data generated with clinical P. aeruginosa SHPHC2, SHPHC15 and SHPHC33, respectively. Experiments were performed in triplicates for each isolate. The percentages of survived bacteria were compared between groups with different pH by using one-way ANOVA with Welch’s test. p value less than 0.05 was considered statistically significant.


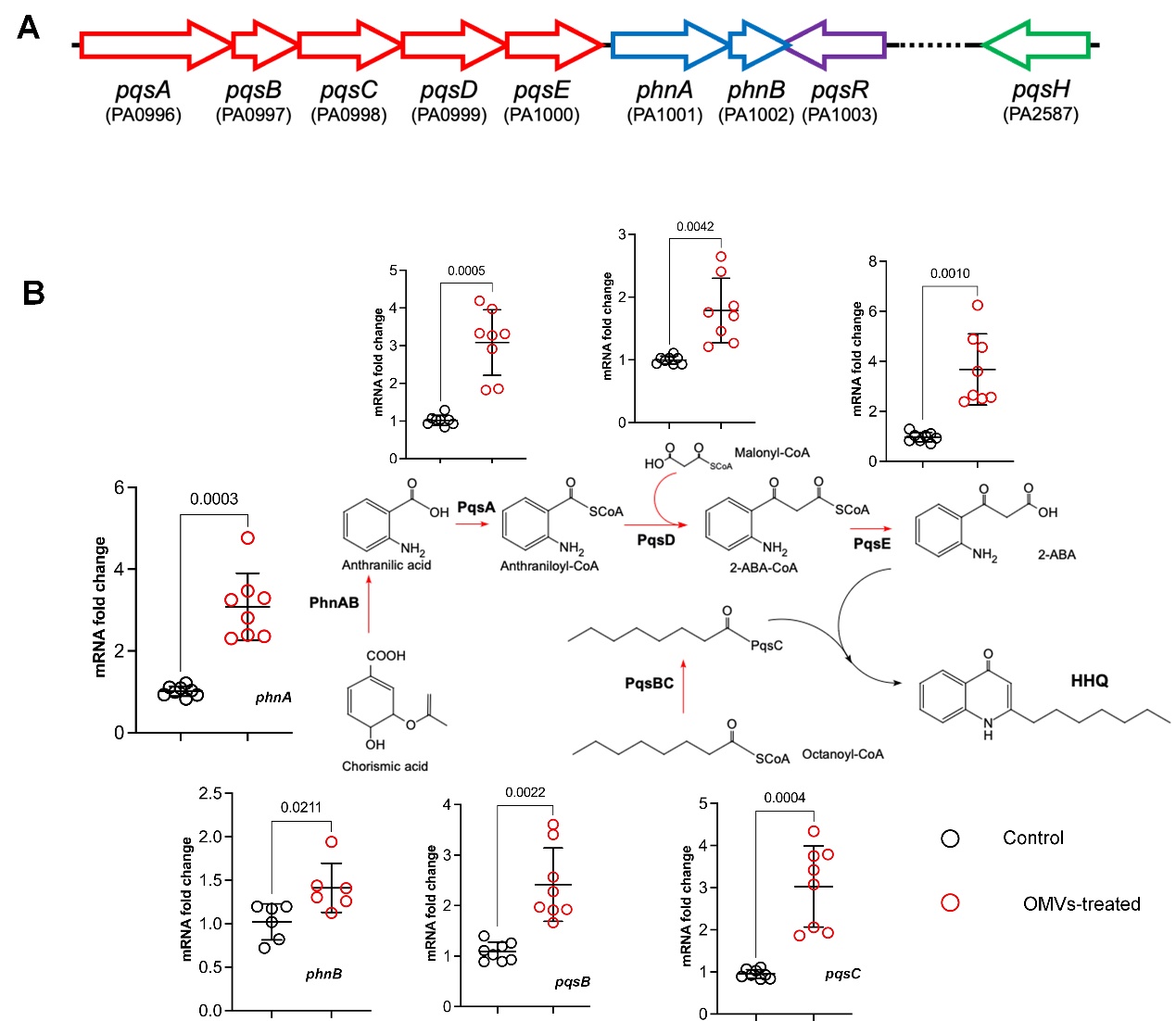


## Figure S4. Genes located in the HHQ biosynthesis cluster were upregulated after the treatment with exogenous OMVs. (A) Genetic organization of the HHQ biosynthesis gene cluster. Transcriptional units (operons) were determined from the RNA sequencing data and illustrated with distinct colors. (B) The functions of proteins encoded by the *pqs* gene cluster in the biosynthesis pathway of HHQ. The adjacent bar charts show the fold change of corresponding gene transcriptions after treatment with OMVs, characterized by quantitative reverse transcription PCR. Comparisons between groups were performed with unpaired Student t test, *p* value less than 0.05 was considered of significance.


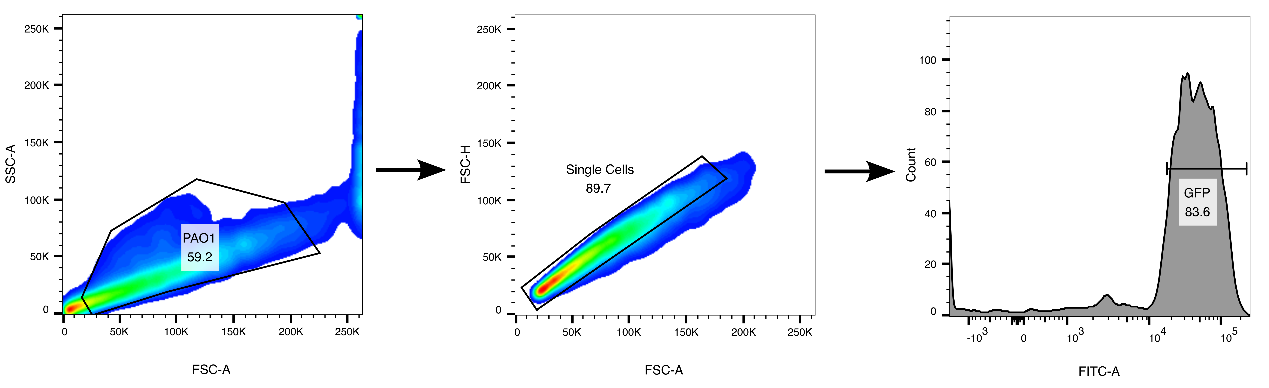


## Figure S5. The gating strategy for the flow cytometry analysis of *P. aeruginosa* bound with fluorescent LL-37.


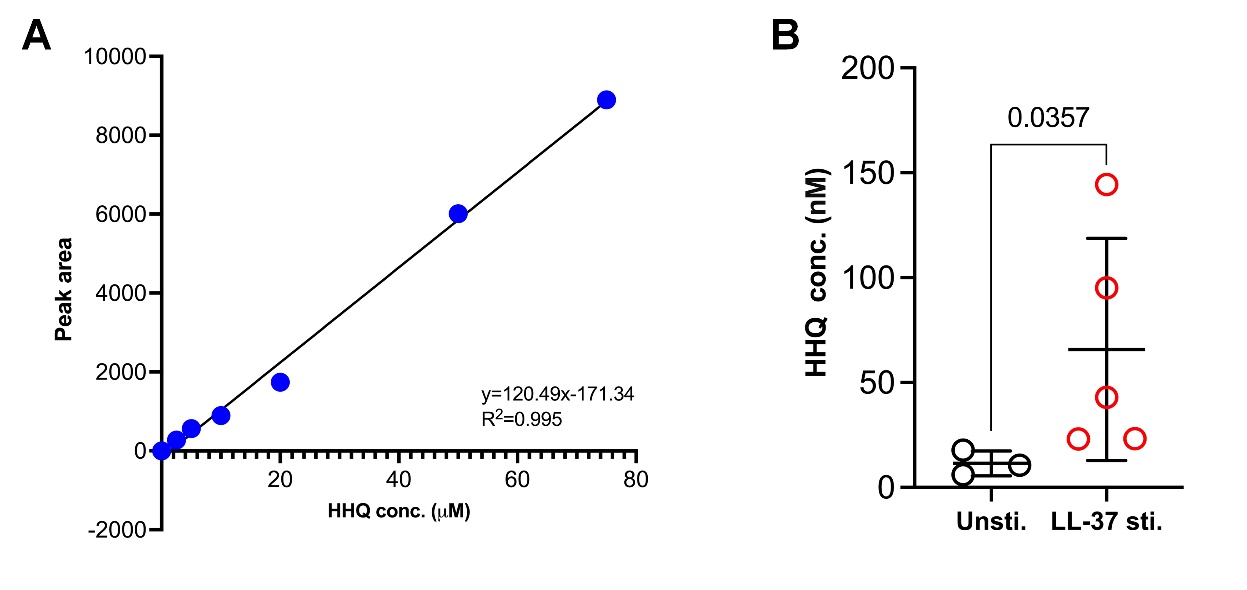


## Figure S6. HHQ in the culture supernatant of *P. aeruginosa* PAO1 increased after treatment stimulation with LL-37 of sub-inhibitory concentration. (A) The fitted standard curve for HHQ quantification. The fitting was performed by linear regression. (B) The calculated HHQ concentration in supernatant assayed by high performance liquid chromatography. Statistical analysis was performed with unpaired Student t test. *p* value less than 0.05 was considered of significance.

**Figure S7. PQS protects *P. aeruginosa* from LL-37 killing.** The error bars plotted indicate the standard error of mean. Student t test was used for statistical analysis in both LL37-treated and untreated groups and the *p* value was shown. *p* value less than 0.05 was considered statistically significant.

**Figure S8. Flow diagram of the study design.** *Pae* is short for *P. aeruginos*a, BE is short for bronchiectasis. Negative in the BE patient groups means no pathogens were isolated by the sputum culture.

**Supplementary Tables**

Table S1. Characteristics of all the participants for BALF LL-37 quantification, related to Figure 1A. (Cohort 1).

| **Characteristic** | **Control (n=15)** | **Bronchiectasis (n=80)** | ***p*** |
| --- | --- | --- | --- |
| Age, years | 59.87±9.97 | 55.51±13.14 | 0.227† |
| BMI, Kg/m2 | 23.79±2.62 | 21.29±3.31 | 0.007† |
| Female | 10(66.7) | 44(55) | 0.403‡ |
| Non-smoking | 4(26.7) | 19(23.8) | 0.753‡ |
| BSI score | NA | 9(4,13) | NA§ |
| Reiff score | NA | 8.5(3,12) | NA§ |
| FVC, L | NA | 2.37±0.82 | NA§ |
| FVC% pred | NA | 72.94±16.08 | NA§ |
| FEV1, L | NA | 1.65±0.72 | NA§ |
| FEV1% pred | NA | 62.07±20.92 | NA§ |
| FEV1/FVC | NA | 69.87±13.32 | NA§ |
| WBC, *10^9^/L | NA | 6.47±1.91 | NA§ |
| Neutrophil % | NA | 59.31±10.24 | NA§ |

†Data were displayed in Mean ± SD. The *p* value was calculated by One-way ANOVA.

‡Data were displayed in n (%). The *p* value was calculated by Chi-squared test.

§Data were displayed in median (IQR). The p value was calculated by Mann-Whitney test.

Abbreviations: BMI: Body Mass Index; BSI: Bronchiectasis Severity Index; FVC, Forced Vital Capacity; FEV1, Forced Expiratory Volume in 1 s; WBC, White Blood Cell.

Table S2. Characteristics of the participants with BE for BALF LL-37 quantification, related to Figure 1A. (Cohort 1).

| **Characteristic** | **Negative (n=32)** | | ***Pae* (n=34)** | **Non-*Pae* (n=14)** | ***p*** |
| --- | --- | --- | --- | --- | --- |
| Age, years | 57.19±13.08 | 52.06±13.03 | | 60.07±12.22 | 0.102† |
| BMI, Kg/m2 | 21.27±3.61 | 21.39±3.19 | | 21.09±3.10 | 0.962† |
| Female | 15 (46.9) | 23 (67.6) | | 6 (42.9) | 0.143‡ |
| Non-smoking | 22 (68.8) | 29 (85.3) | | 10 (71.4) | 0.258‡ |
| BSI score | 4.5 (3.25, 8.5) | 13 (11,15.25) | | 5.5 (3, 12.25) | <0.001§ |
| Reiff score | 5 (2, 9) | 12 (9, 14) | | 4 (2, 6.75) | <0.001§ |
| FVC, L | 2.42±0.14 | 2.26±0.17 | | 2.50±0.24 | 0.630† |
| FVC% pred | 75.1±2.8 | 70.0±3.2 | | 75.8±4.4 | 0.363† |
| FEV1, L | 1.73±0.13 | 1.54±0.14 | | 1.72±0.20 | 0.558† |
| FEV1/FVC | 70.4±2.5 | 69.6±2.1 | | 69.3±4.5 | 0.960† |
| WBC, *10^9^/L | 6.46±0.40 | 6.79±0.38 | | 5.71±0.37 | 0.291† |
| Neutrophil % | 61.3±2.3 | 60.9±1.6 | | 50.8±2.1 | 0.008† |

†Data were displayed in Mean ± SD. The *p* value was calculated by One-way ANOVA.

‡Data were displayed in n (%). The *p* value was calculated by Chi-squared test.

§Data were displayed in median (IQR). The p value was calculated by Mann-Whitney test.

Abbreviations: BMI: Body Mass Index; BSI: Bronchiectasis Severity Index; FVC, Forced Vital Capacity; FEV1, Forced Expiratory Volume in 1 s; WBC, White Blood Cell.

**Table S2.** Characteristics of the participants for sputum LL-37 quantification, related to Figure 1E-G (Cohort 2).

| **Characteristic** | **Negative (n=18)** | ***Pae* (n=9)** | **Non-*Pae* (n=6)** | ***p*** |
| --- | --- | --- | --- | --- |
| Age, years | 56.44±17.12 | 53.11±15.70 | 67.17±8.34 | 0.229† |
| BMI, Kg/m2 | 21.33±3.12 | 21.81±2.98 | 20.88±2.80 | 0.850† |
| Female | 11(61.1) | 5(55.6) | 4(66.7) | 1.000‡ |
| Non-smoking | 4(22.2) | 4(22.2) | 2(33.3) | 0.867‡ |
| BSI score | 10.5(1.75,12) | 14.5(12.5,15) | 9(4.5,12.25) | 0.004§ |
| Reiff score | 4(4,9.75) | 8(7,18) | 8.5(5.5,13.25) | 0.145§ |
| FVC, L | 2.17±0.89 | 1.86±0.56 | 2.17±0.61 | 0.624† |
| FVC% pred | 70.7±21.57 | 64.55±14.89 | 74.35±18.31 | 0.624† |
| FEV1, L | 1.54±0.82 | 1.26±0.47 | 1.70±0.40 | 0.455† |
| FEV1% pred | 60.91±25.83 | 54.38±19.80 | 74.18±18.48 | 0.289† |
| FEV1/FVC | 68.96±15.73 | 67.97±15.03 | 80.19±11.78 | 0.253† |
| WBC, *10^9^/L | 6.36±3.09 | 7.39±1.92 | 5.52±1.86 | 0.421† |
| Neutrophil % | 64.22±9.56 | 65.73±7.44 | 58.93±11.42 | 0.377† |

†Data were displayed in Mean ± SD. The *p* value was calculated by One-way ANOVA.

‡Data were displayed in n (%). The *p* value was calculated by Chi-squared test.

§Data were displayed in median (IQR). The p value was calculated by Mann-Whitney test.

Abbreviations: BMI: Body Mass Index; BSI: Bronchiectasis Severity Index; FVC, Forced Vital Capacity; FEV1, Forced Expiratory Volume in 1 s; WBC, White Blood Cell.

Table S3. Characteristics of all the participants for sputum lactate quantification, related to Fig 2A (Cohort 3).

| **Characteristic** | **Control (n=30)** | **Bronchiectasis (n=26)** | ***p*** |
| --- | --- | --- | --- |
| Age, years | 50.50±10.87 | 51.35±12.61 | 0.788† |
| BMI, Kg/m2 | 22.08±2.04 | 22.92±2.75 | 0.197† |
| Female | 20 (66.7) | 19 (73.1) | 0.603‡ |
| Non-smoking | 22 (73.3) | 20 (76.9) | 0.757‡ |
| BSI score | NA | 7(4,10) | NA§ |
| Reiff score | NA | 9.5(4.75,14) | NA§ |
| FVC, L | NA | 2.47±1.17 | NA† |
| FVC% pred | NA | 73.2±21.5 | NA† |
| FEV1, L | NA | 1.80±0.92 | NA† |
| FEV1% pred | NA | 64.7±22.8 | NA† |
| FEV1/FVC | NA | 72.2±9.7 | NA† |
| WBC, *10^9^/L | NA | 6.82±2.73 | NA† |
| Neutrophil % | NA | 64.0±11.4 | NA† |

†Data were displayed in Mean ± SD. The *p* value was calculated by One-way ANOVA.

‡Data were displayed in n (%). The *p* value was calculated by Chi-squared test.

§Data were median (IQR). The p value was calculated by Mann-Whitney test.

Abbreviations: BMI: Body Mass Index; BSI: Bronchiectasis Severity Index; FVC, Forced Vital Capacity; FEV1, Forced Expiratory Volume in 1 s; WBC, White Blood Cell; NA, Not Available.

Table S4. Characteristics of the BE patients for sputum lactate quantification, related to Figure 2B (Cohort 3)

| **Characteristic** | **Negative (n=7)** | ***Pae* (n=15)** | **Non-*Pae* (n=4)** | ***p*** |
| --- | --- | --- | --- | --- |
| Age, years | 55.43±17.16 | 49.60±11.75 | 50.75±6.13 | 0.616^a^ |
| BMI, Kg/m2 | 21.34±2.09 | 23.02±2.16 | 25.31±4.37 | 0.063^a^ |
| Female | 6 (85.7) | 11 (73.3) | 2 (50) | 0.505^b^ |
| Non-smoking | 6 (85.7) | 11 (73.3) | 3 (75) | 1.000^b^ |
| BSI score | 7 (3,9) | 8 (5,13) | 4 (3.5, 5.5) | 0.190^c^ |
| Reiff score | 5 (4,6) | 11 (9,16) | 5 (1.75,10.5) | 0.035^c^ |
| FVC, L | 2.55±0.34 | 2.31±0.37 | 2.94±0.67 | 0.709^a^ |
| FVC% pred | 82.3±6.0 | 65.8±6.3 | 83.7±10.9 | 0.174^a^ |
| FEV1, L | 1.91±0.25 | 1.64±0.29 | 2.25±0.59 | 0.571^a^ |
| FEV1% pred | 75.5±6.3 | 56.1±6.5 | 76.8±12.5 | 0.115^a^ |
| FEV1/FVC | 74.7±2.9 | 70.1±3.1 | 75.3±2.8 | 0.529^a^ |
| WBC, *10^9^/L | 6.55±0.98 | 7.10±0.79 | 6.00±0.76 | 0.793^a^ |
| Neutrophil % | 63.2±4.7 | 66.1±3.0 | 55.8±4.4 | 0.368^a^ |

^a^ Data were displayed in Mean ± SD. The *p* value was calculated by One-way ANOVA.

^b^ Data were n (%). The *p* value was calculated by Chi-squared test.

^c^ Data were median (IQR). The p value was calculated by Mann-Whitney test.

Abbreviations: BMI: Body Mass Index; BSI: Bronchiectasis Severity Index; FVC, Forced Vital Capacity; FEV1, Forced Expiratory Volume in 1 s; WBC, White Blood Cell.

Table S5. *P. aeruginosa* isolates used in this study.

| **Name** | **Time of isolation** | **Source** |
| --- | --- | --- |
| SHPHC2 | Jan. 2020 | Isolated from **BALF** of a male BE patient born in 1945. *P. aeruginosa* was isolated from the sputum of this patient in Dec. 2018 and Dec. 2019. |
| SHPHC15 | Jun. 2020 | Isolated from **sputum** of a male BE patient born in 1955. *P. aeruginosa* was isolated from the sputum of this patient in Feb. and Jun. 2020. |
| SHPHC33 | Mar. 2021 | Isolated from **BALF** of female BE patient born in 1958. *P. aeruginosa* was isolated from the sputum of this patient in Dec. 2017, Sept. 2019 and Mar. 2021. |
| PAO1 | / | Lab archive |

Table S6. Primers used in this study.

| **Primer Name** | **Sequence (5' to 3')** | **Amplicon size (bp)** | **Purpose** |
| --- | --- | --- | --- |
| pqsA-qF | TGCCGGACCTACATTCTCTC | 151 | Quantification of the *pqsA* gene transcription by qRT-PCR |
| pqsA-qR | GATTGATCACGGCGGGAATG |  |  |
| pqsB-qF | ATCCGTTGCAGAAGGTCCTC | 171 | Quantification of the *pqsB* gene transcription by qRT-PCR |
| pqsB-qR | ACTCGCTGTCCACTTCCAAT |  |  |
| pqsC-qF | GGACATGCTGATCTGTTCGG | 151 | Quantification of the *pqsC* gene transcription by qRT-PCR |
| pqsC-qR | CATCTGCGAATCCAATGGCA |  |  |
| pqsD-qF | ACCTTCCTCGACGAGAATGT | 172 | Quantification of the *pqsD* gene transcription by qRT-PCR |
| pqsD-qR | GGTTCGGTTGATGGCAGATC |  |  |
| pqsE-qF | CTGGATGATGACCTGTGCCT | 191 | Quantification of the *pqsE* gene transcription by qRT-PCR |
| pqsE-qR | TCGTAGTGCTTGTGGGTGAT |  |  |
| phnA-qF | GATGCGGAACTGGACAATCG | 158 | Quantification of the *phnA* gene transcription by qRT-PCR |
| phnA-qR | TAGCGATCGACCTTGAGCAT |  |  |
| phnB-qF | GTCCCTGCGTTTCGATCAG | 192 | Quantification of the *phnB* gene transcription by qRT-PCR |
| phnB-qR | ATCGACTCGGGATGGAACTG |  |  |
| rpoD-qF | CGATCGGTGACGACGAAGAT | 176 | Used as the reference gene for the relative quan-tification of gene transcription in *P. aeruginosa* |
| rpoD-qR | GTTCATGTCGATGCCGAAGC |  |  |

**References**

1. Tjaden B. A computational system for identifying operons based on RNA-seq data. *Methods*. 2020;176:62-70.

2. Winsor GL, Griffiths EJ, Lo R, Dhillon BK, Shay JA, Brinkman FS. Enhanced annotations and features for comparing thousands of Pseudomonas genomes in the Pseudomonas genome database. *Nucleic Acids Res*. 2016;44(D1):D646-53.
